# Academic stress and academic engagement among Peruvian university students during their first experience with online learning

**DOI:** 10.3389/fpsyg.2026.1859567

**Published:** 2026-07-16

**Authors:** Lizley Janne Tantalean-Terrones, Jimmy Oscar Callohuanca-Aceituno

**Affiliations:** 1 Faculty of Psychology, Federico Villarreal National University, Lima, Peru; 2 Faculty of Economics and Planning, La Molina National Agrarian University, Lima, Peru

**Keywords:** academic engagement, academic stress, COVID-19, online learning, university students

## Abstract

**Introduction:**

The transition to fully online learning during the COVID-19 health emergency affected students’ academic experiences. This study aimed to examine academic stress and academic engagement in 527 Peruvian university students during their first experience with online learning.

**Methods:**

Using a non-experimental, quantitative, cross-sectional, and correlational design, the study employed the adapted SISCO Inventory of Academic Stress and the Utrecht Engagement Scale for Students.

**Results:**

The results showed that 44.2% of students reported high levels of stress symptoms, 55.6% reported high levels of stressors, 65.5% reported high levels of coping strategies, and 42.3% reported high levels of academic engagement. Significant differences were found by sex, age, perceived learning achievement, and occupational status, but not by degree program or difficulties accessing virtual classes. Academic engagement and its dimensions were positively correlated with coping strategies and negatively correlated with stress symptoms but were not significantly correlated with stressors. Finally, the structural model showed that stressors, coping strategies, and academic engagement were jointly associated with 37.5% of the variance in stress symptoms.

**Discussion:**

In addition, coping strategies were positively associated with academic engagement, and academic engagement showed a weak mediation effect, particularly in the association between coping strategies and stress symptoms.

## 1 Introduction

The declaration of COVID-19 as a pandemic by the World Health Organization (WHO) brought about structural changes worldwide and imposed a new way of life aimed at protecting individual and collective health. This situation accelerated the digitalization of public and private services and exposed major shortcomings in the digital literacy of the stakeholders involved (Organización Panamericana de la Salud, 2020).

In this context, public education in Peru, unable to provide in-person services, implemented the strategy “Aprendo en casa” as a temporary solution for regular and alternative basic education (Ministerio de Educación, 2020). Its implementation lasted for 2 years as the pandemic worsened and conditions were developed for a blended educational environment (Gobierno del Perú, 2021). In higher education, meanwhile, the exclusive delivery of classes in a virtual format represented a significant departure from the provisions of the university law then in force (Law No. 30220, Art. 47, 2014).

These circumstances prompted government measures enabling public universities to manage the implementation and delivery of educational services based on their available budgets. These measures included strategies to ensure continuity in academic training, such as implementing educational platforms for videoconferencing, assignment submission, and assessment; training teachers to facilitate learning using these resources; and training students as users. Measures were also taken to reduce the gap in internet connectivity among students living in poverty. These included the acquisition and distribution of modems, the redesign of benefits for high-achieving students, and the creation of the continuity scholarship to prevent student dropout (Figallo et al., 2020).

Accordingly, Peru’s public universities began their activities while navigating organizational, procedural, communication, and decision-making challenges in a completely unfamiliar context. These challenges were compounded by bureaucratic bottlenecks in budget allocation, the need to adapt curricula, and the socioemotional strain on staff during the health crisis. Limited digital literacy, lack of technological devices, and poor internet connectivity also became obstacles that were largely overlooked in the rush to reactivate higher education services (Miguel, 2020).

These conditions tested the leadership and administrative capacity of university authorities as faculty and students transitioned to online learning, including the need to address academic and socioemotional problems (Sánchez, 2022). As a result, the start of the first semester of 2020 was delayed by 4–5 months—equivalent to one academic term—and universities that failed to hold admissions examinations in 2020 lost incoming students, deepening inequities in access to higher education among lower-income populations (Bazán-Ramírez et al., 2020).

In this context, university students were already immersed in a demanding educational process when they were abruptly required to shift to an online environment. In this setting, meaningful learning and the achievement of professional competencies depended heavily on personal organization and self-discipline. The absence of these capacities fostered academic stress as a result of failure, dissatisfaction, and educational burnout (González, 2020). This became even more pronounced when rigorous academic demands were combined with difficulties adapting to virtual learning, exacerbated by a lack of resources and tools for optimal performance (Osorio et al., 2020).

Accordingly, academic stress emerged as students’ response to the demands of the educational environment when those demands exceeded their capacities or resources. Such stress may arise from external contingencies—such as tight deadlines, heavy coursework, and evaluations—as well as from individual factors such as self-imposed demands. Its effects extend to learning outcomes and to physical and mental health, especially in cognitive, emotional, and behavioral domains (Benítez-Agudelo et al., 2025; Bernardo et al., 2025; Pozos-Radillo et al., 2014). It may even lead students to perceive the educational environment as threatening, triggering fear or anxiety about failure (Zhang et al., 2024).

Under these conditions, strong engagement in academic activities mobilizes affect and behavior (Kahn, 1990). It also promotes students’ achievement orientation in response to existing challenges (Cavazos and Encinas, 2016) and entails high levels of concentration, effort, energy, and inspiration to meet the demands of the educational process (Liébana-Presa et al., 2018). In addition, it is reflected in a positive attitude and proactive behavior toward the fulfillment of academic tasks (Tortosa et al., 2022). However, this engagement was significantly affected by the transition to online learning during the health emergency (Marenco et al., 2024).

Academic engagement is understood as the outcome of the personal resources of vigor, dedication, and absorption that students mobilize in response to their educational responsibilities. It entails sustained participation and investment in learning, reflected in ongoing cognitive effort, motivated by deep involvement in the educational process (Metu, 2024; Peker, 2024). It also constitutes a positive psychological state that enhances the achievement of learning goals and supports effective academic performance (Karki et al., 2020; Salanova et al., 2010).

Academic stress and academic engagement have been examined together in a variety of contexts. Among Croatian youth, academic demands and emotional exhaustion were found to be determinants of stress, but not of academic engagement or its components; women with higher dedication and absorption also showed a greater tendency toward stress (Rončević and Marinić, 2024). Among U. S. students, stress has been associated with academic performance, with academic engagement playing a mediating role in this relationship (Tormon et al., 2023). Other studies have documented the negative association between stress and academic engagement, as well as with mental health (Saleem et al., 2022; Van Ryzin et al., 2022). In Spanish students, a strong negative correlation between stress and academic engagement has been reported (Arrivillaga et al., 2022). Among Turkish adolescents, stress levels were found to be mediated by sociodemographic factors (Simsek and Günay, 2023). In the sports-work setting, in turn, greater task engagement has been associated with lower stress levels (Barbado and Martínez-Moreno, 2020), while pride in one’s work helps sustain high engagement despite the stress generated by complex task conditions (Saleem et al., 2022).

Accordingly, the present study analyzed academic stress and academic engagement in students from two public universities from descriptive, comparative, correlational, and structural-modeling perspectives. Specifically, it described both variables in terms of the levels reached by students; compared the variables and their dimensions overall and by sex, year of study, difficulties accessing virtual classes, perceived learning achievement, degree program, and occupational status; examined correlations between the variables and their dimensions; and developed a structural equation model including the study variables and their dimensions.

Examining academic stress and academic engagement is especially relevant in the context of the health emergency and virtual learning, as students were required to adapt to a completely new learning experience using their own resources. Analyzing these variables in relation to different sociodemographic conditions and their associations makes it possible to draw valuable conclusions for stratified interventions based on students’ needs and highlights academic engagement as a resilience-promoting factor in situations that threaten university students’ personal and professional development goals.

## 2 Method

### 2.1 Study design

This study used a non-experimental, quantitative, cross-sectional, and correlational design (Hernández-Sampieri et al., 2014). It was complemented by descriptive and comparative analyses (Sambrano, 2020) and included structural equation modeling to examine the relationships among the study variables and their dimensions and to evaluate direct, indirect, and total effects in statistical terms, without assuming definitive causal inferences (Kline, 2016).

### 2.2 Participants

A total of 527 participants were recruited from a population of 2012 students at two public universities in Metropolitan Lima: 282 students from the Faculty of Psychology and 245 engineering students. The sample included 352 women and 175 men aged 18–33 years (*M* = 22.9, SD = 3.09). All participated voluntarily, and the sample was non-probabilistic, selected using the snowball technique. It should be noted that this sampling approach facilitated access to participants and increased their availability, while also expanding the scope of data collection; however, it may have introduced selection bias and limited the representativeness of the sample.

### 2.3 Instruments

#### 2.3.1 Sociodemographic data sheet

Questionnaire including questions on sex, age range, occupational status, year of study, number of devices used to access classes, barriers encountered during participation in virtual classes, perceived difficulty using the educational platform, and perceived learning achievement during the current semester.

#### 2.3.2 SISCO inventory of academic stress V (adapted)

The original instrument consists of a 45-item scale with three dimensions—stressors, symptoms, and coping strategies (15 items per dimension)—and a six-point Likert response scale ranging from lower to higher intensity (Barraza-Macías, 2007). The instrument was adapted for virtual learning during COVID-19 and included two items in the strategies dimension: “browsing the internet” and “using video games.” It was evaluated by expert judgment on the criteria of clarity, objectivity, currency, organization, sufficiency, relevance, consistency, coherence, methodology, and applicability, with Aiken’s V values ranging from 0.85 to 1.00. It was subsequently administered to 151 Peruvian university students and yielded Cronbach’s alpha coefficients above 0.88 and item-test correlations above 0.80 for all three dimensions (Alania et al., 2020). The aforementioned study did not present evidence of a structural model in which the factors converge into a general variable, because the strategies dimension reflects coping responses rather than uncontrolled stress.

It should be noted that the instrument’s construct validity criteria were updated in the present sample through confirmatory factor analysis, which showed adequate fit indices: χ^2^(1028) = 4343.80, χ^2^/df = 4.23, CFI = 0.960, TLI = 0.958, RMSEA = 0.078, and SRMR = 0.070. Although PClose was significant (*p* < 0.001), indicating the absence of close fit in the strict sense, the incremental and residual indices showed an overall acceptable model fit. Convergent validity was also assessed using the AVE index, which yielded moderate values, whereas discriminant validity was supported by HTMT indices below 0.85. In addition, the reliability of the inventory and its dimensions was supported by *α* and *ω* coefficients above 0.826, indicating adequate internal consistency.

#### 2.3.3 Utrecht engagement scale for students (UWES-17)

The instrument was subjected to validity and reliability analyses in 1,585 Chilean university students, showing favorable internal consistency, with Cronbach’s alpha values ranging from 0.88 to 0.90, as well as adequate model fit for a three-factor structure estimated by maximum likelihood (Cruzat, 2020). In Peruvian university students, the three-factor model was likewise confirmed, with adequate factorial fit indices; the study reported reliability coefficients of *α* < 0.74 for the total scale and its components (Tacca-Huamán et al., 2021).

In this study, basic validity and reliability indicators were updated. As evidence of construct validity, item-test correlations above 0.529 were identified for the total scale and above 0.574 for absorption, 0.722 for dedication, and 0.563 for vigor. Regarding reliability, α and *ω* coefficients were above 0.919 for the total scale and above 0.810 for absorption, 0.928 for dedication, and 0.943 for vigor. The differences between the alpha values obtained in the present assessment and those reported by Tacca-Huamán et al. (2021) may be explained by differences in sample characteristics.

In addition, the UWES-17 factor model showed inadequate fit to the sample data: χ^2^(116) = 1007.36, χ^2^/df = 8.68, CFI = 0.924, TLI = 0.911, RMSEA = 0.121, SRMR = 0.059, and PClose < 0.001. Even so, its three-factor structure was retained for reasons of theoretical consistency, and engagement was interpreted as an integrated construct within the structural model.

### 2.4 Procedure

The instruments were administered through an online Outlook form, and the evaluation link was distributed for 2 months among psychology students at Federico Villarreal National University and students from the Business Management Engineering School at La Molina National Agrarian University, before the midterm evaluation period at both institutions.

### 2.5 Data analysis

Stress and academic engagement levels were described through frequencies and percentages in order to characterize the distribution of the main variables. A comparative analysis was then conducted according to participants’ sociodemographic characteristics, using the Mann–Whitney U and Kruskal-Wallis tests; in addition, correlational analyses were conducted between the main variables and their dimensions using Spearman’s rho coefficient. It should be noted that the nonparametric tests were selected because the data did not meet the normality assumption; see the Supplementary material.

Finally, a structural equation model was specified in which stress symptoms were specified as the endogenous variable, stressors and coping strategies were specified as exogenous variables, and academic engagement was modeled as a mediator. The model was estimated using the Robust Maximum Likelihood (MLR) method because the variables vigor, absorption, dedication, symptoms, strategies, and stressors did not meet the assumption of multivariate normality, as indicated by significant deviations according to Mardia’s coefficient (b2d = 53.269; *z* = 6.172; *p* < 0.001). Another factor supporting the use of this estimator was the five-point scalar measurement of the variables, which allowed them to be treated as ordinal values with a continuous approximation. In addition, the Maximum Likelihood (ML) estimator was used solely to calculate the direct, indirect, and total effects of the mediation model, given the need to apply the bootstrap method for that purpose. It should be noted that data processing was carried out using SPSS version 29 and RStudio version 4.5.2.

## 3 Results

### 3.1 Descriptive analysis

The descriptive analysis showed that 44.2 and 55.6% of the students presented high levels of stress symptoms and stressors, respectively. Likewise, 65.5% showed high levels of coping strategies and 42.3% showed high levels of academic engagement (see Table 1).

**Table 1:** Levels of academic engagement and factors of academic stress in university students (*n* = 527).

Variable/factor	Level	*f*	*%*	Variable	Level	*f*	*%*
Engagement	Low	28	5.3	Stressors	Low	30	5.7
	Moderate	276	52.4		Moderate	204	38.7
	High	223	42.3		High	293	55.6
Symptoms	Low	82	15.6	Coping strategies	Low	4	0.8
	Moderate	212	40.2		Moderate	178	33.8
	High	233	44.2		High	345	65.5

Source: prepared by the authors.

### 3.2 Comparative analysis

Comparisons of the factors of academic stress and academic engagement (overall and by dimension) by sex showed significant differences, with women reporting higher levels of stressors and stress symptoms than men (*p* < 0.002). The effect size was small for stress symptoms (see Table 2).

**Table 2:** Comparison of academic stress factors and academic engagement (construct and dimensions) by sex.

Sex	*df*	Stress					Academic engagement				
		Variable/ Dimension	Rank	*U*	*p*	*r*	Variable/ Dimension	*Rank*	*U*	*p*	*r*
Men/ Women	1	Stressors	−46.81	25328.500	0.001*	0.145	Total	−57.99	24021.500	<0.001*	0.179
	1	Symptoms	−75.74	21946.000	< 0.001*	0.234	Vigor	−44.6	25587.500	0.002*	0.138
	1	Coping strategies	−24.84	27897.000	0.078	0.077	Dedication	−58.49	23962.500	<0.001*	0.181
	1						Absorption	−60.17	23767.500	<0.001*	0.186

Source: prepared by the authors.

By age range, differences were observed in stressors and symptoms among students aged 21 to 24 years. A similar result was found for dedication among students aged 25 years and older (*p* < 0.027), although the effect size was negligible (η^2^_h_ < 0.20; see Table 3).

**Table 3:** Comparison of academic stress factors and academic engagement (construct and dimensions) by age.

Age range	*df*	Stress					Academic engagement				
		Variable/ Dimension	Rank	*H*	*p*	*η^2^_h_*	Variable/ Dimension	*Rank*	*H*	*p*	*η^2^_h_*
18–20	2	Stressors	250.98	9.031	0.011*	0.010	Total	257.01	4.952	0.084	0.002
21–24			280.34					251.50			
25 and above			233.95					300.62			
18–20	2	Symptoms	255.65	10.135	0.006*	0.012	Vigor	258.78	4.719	0.094	0.001
21–24			283.31					253.93			
25 and above			222.84					293.17			
18–20	2	Coping strategies	263.51	2.127	0.345	−0.004	Dedication	261.51	7.243	0.027*	0.006
21–24			255.32					247.73			
25 and above			285.92					306.36			
18–20	2						Absorption	250.85	4.303	0.116	0.001
21–24								255.32			
25 and above								296.10			

Source: prepared by the authors.

Regarding difficulties accessing virtual classes, no significant differences were observed in any of the comparative analyses (*p* > 0.05). In addition, with respect to perceived learning achievement, participants showed highly significant differences in stressors, stress symptoms, coping strategies, and overall academic engagement and its dimensions, with U > 23790.500 and *p* = 0.001, such that greater perceived learning was associated with lower stress-related factors and higher academic engagement, although the effect sizes were small to approaching moderate (see Table 4).

**Table 4:** Comparison of academic stress factors and academic engagement (construct and dimensions) by difficulty accessing virtual classes and perceived learning achievement.

Group	*df*	Stress					Academic engagement				
		Variable/ Dimension	Rank	*U*	*p*	*r*	Variable/ Dimension	Rank	*U*	*p*	*r*
Difficulty accessing virtual classes (Yes / No)	1	Stressors	29.89	26542.500	0.062	0.081	Total	6.630	24438.000	0.679	0.018
	1	Symptoms	4.710	24264.000	0.769	0.013	Vigor	4.850	24276.500	0.762	0.013
	1	Coping strategies	−8.100	23105.000	0.613	−0.022	Dedication	−0.260	24276.500	0.762	−0.001
	1						Absorption	12.11	24933.500	0.449	0.033
Perceived learning achievement (Limited / Sufficient)	1	Stressors	70.05	23960.000	0.001*	−0.223	Total	−82.02	42794.000	0.001*	0.261
	1	Symptoms	71.42	23790.500	0.001*	−0.227	Vigor	−78.84	42401.500	0.001*	0.251
	1	Coping strategies	−62.68	40399.500	0.001*	0.200	Dedication	−82.04	42797.000	0.001*	0.262
	1						Absorption	−70.09	41317.000	0.001*	0.223

Source: prepared by the authors.

With respect to the degree program, significant differences were observed only in the dedication dimension, with higher scores among psychology students. A similar pattern was found for the use of stress-coping strategies, overall academic engagement, and the vigor and dedication dimensions among students who combined study with paid work (*p* < 0.044), although the effect size was negligible across contrasts (*r* < 0.20; see Table 5).

**Table 5:** Comparison of academic stress factors and academic engagement (construct and dimensions) by degree program and occupational status.

Group	*df*	Stress				Academic engagement					
		Variable/ Dimension	Rank	*U*	*p*	*r*	Variable/ Dimension	Rank	*U*	*p*	*r*
Degree program (Engineering / Psychology)	1	Stressors	−22.22	31633.000	0.095	0.073	Total	−17.59	32239.000	0.186	0.075
	1	Symptoms	−15.71	32485.000	0.237	0.051	Vigor	−2.36	34235.500	0.859	0.008
	1	Coping strategies	−8.21	33468.500	0.537	0.027	Dedication	−35.14	29938.500	0.008*	0.115
	1						Absorption	−12.28	32934.500	0.355	0.040
Occupation (Only studying / Studying and working)	1	Stressors	8.92	31072.000	0.518	−0.028	Total	−27.76	28772.000	0.044*	0.088
	1	Symptoms	23.49	29293.000	0.088	−0.074	Vigor	−28.8	28644.500	0.036*	0.091
	1	Coping strategies	−28.54	28676.500	0.038*	0.090	Dedication	−29.18	28599.000	0.034*	0.092
	1						Absorption	−24.45	29176.500	0.076	0.077

Source: prepared by the authors.

### 3.3 Correlational analysis

In addition, academic engagement and its dimensions showed a moderate positive correlation with the coping strategies factor, a weak negative correlation with stress symptoms (*p* < 0.001), and no significant correlation with stressors (*p* > 0.05; see Table 6).

**Table 6:** Correlations between academic engagement, its dimensions, stress symptoms, stressors, and coping strategies.

Variable	*Rho*	*Sig.*	Variable	*Rho*	*Sig.*
Engagement–symptoms	−0.166**	0.000	Dedication - Symptoms	−0.161**	0.000
Engagement–stressors	−0.061	0.161	Dedication - Stressors	−0.081	0.062
Engagement–coping strategies	0.479**	0.000	Dedication - Coping strategies	0.434**	0.000
Absorption–symptoms	−0.107*	0.014	Vigor - Symptoms	−0.190**	0.000
Absorption–stressors	−0.010	0.819	Vigor - Stressors	−0.080	0.068
Absorption–coping strategies	0.451**	0.000	Vigor - Coping strategies	0.438**	0.000

**p < 0.01; *p < 0.05.

### 3.4 Modeling of the variables

**Figure 1:**
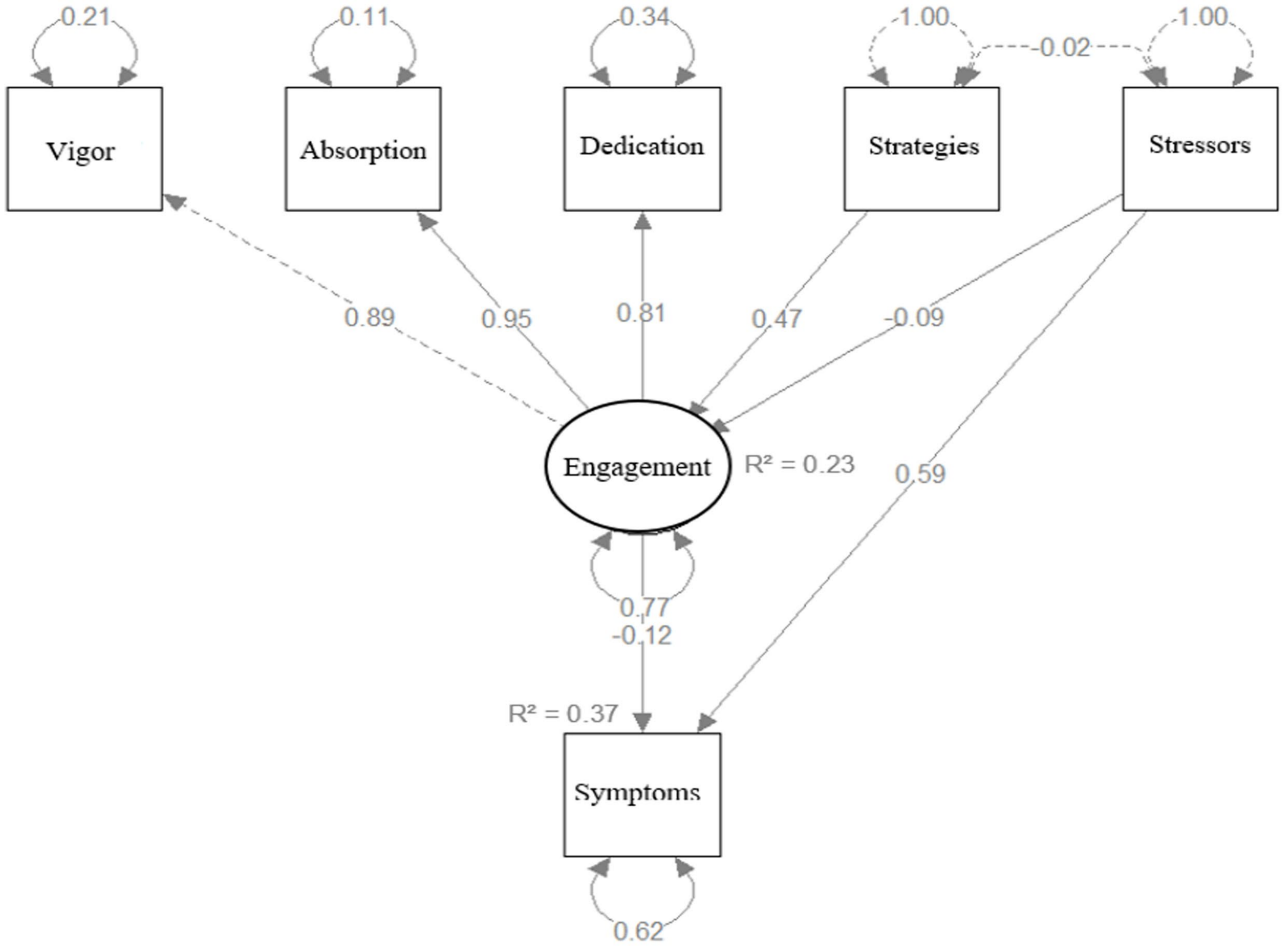
Structural model of the relationships among the study variables.

In the structural model, academic engagement was analyzed as a latent construct represented by vigor, absorption, and dedication, whereas stressors, coping strategies, and stress symptoms were included as stress-related components. In this model, stress symptoms were treated as the endogenous variable, stressors and coping strategies as exogenous variables, and academic engagement as the mediating variable between stressors and stress symptoms. Under these specifications, the structural model showed that the variables included were associated with 37.5% of the variance in stress symptoms, while academic engagement was negatively associated with stress symptoms (−0.12), with greater engagement being, with fewer symptoms. Academic engagement was also well represented by its dimensions (vigor, absorption, and dedication; see Figure 1).

It should be noted that the latent engagement construct (vigor, absorption, and dedication) showed internal consistency (CR = 0.914) and convergent validity (AVE = 0.781), in accordance with the criteria proposed in the literature (Fornell and Larcker, 1981).

In addition, the model showed adequate global fit indices (χ^2^/df = 3.185, CFI = 0.988, TLI = 0.976, RMSEA = 0.069, SRMR = 0.024, and PClose = 0.073), with coefficients within acceptable ranges. It should be noted that CFI and TLI values above 0.95 indicate excellent incremental fit, whereas SRMR and RMSEA values below 0.08 suggest acceptable fit of the structural model (see Table 7).

**Table 7:** Fit indices for the structural model.

Model	CMIN	df	CMIN/df	PClose	CFI	TLI	RMSEA	SRMR
Structural model	22.299	7	3.185	0.073	0.988	0.976	0.069	0.024

CMIN = chi-square minimum; df = degrees of freedom; CMIN/df = normed chi-square; CFI = Comparative Fit Index; TLI = Tucker-Lewis Index; RMSEA = Root Mean Square Error of Approximation; SRMR = Standardized Root Mean Square Residual; PClose = RMSEA test of close fit.

Finally, in the mediation model, coping strategies showed a positive and significant association with academic engagement (*β* = 0.471, *p* < 0.001), whereas stressors showed a marginal negative relationship (*β* = −0.085, *p* = 0.055). Academic engagement was associated with fewer symptoms (*β* = −0.118, *p* = 0.003), whereas stressors were associated with an increase in symptoms (*β* = 0.590, *p* < 0.001). Regarding indirect effects, coping strategies were significantly associated with symptoms through academic engagement (*β* = −0.055, *p* = 0.004), whereas the indirect effect of stressors did not reach significance (*β* = 0.010, *p* = 0.097). Overall, the total effects showed that coping strategies were associated with a reduction in symptoms (*β* = −0.055, *p* = 0.004) and stressors with an increase in symptoms (*β* = 0.600, *p* < 0.001; see Table 8).

**Table 8:** Indirect and total mediation effects of the model.

Effect	*B*	*β*	SE	*z*	*p*	95% CI
Direct (Coping strategies → Engagement)	0.265	0.471	0.024	10.925	< 0.001	[0.218, 0.312]
Direct (Stressors → Engagement)	−0.041	−0.085	0.022	−1.916	0.055	[−0.084, 0.001]
Direct (Engagement → Symptoms)	−0.304	−0.118	0.101	−3.007	0.003	[−0.499, −0.108]
Direct (Stressors → Symptoms)	0.741	0.590	0.043	17.435	< 0.001	[0.656, 0.824]
Indirect (Coping strategies → Symptoms)	−0.081	−0.055	0.028	−2.906	0.004	[−0.135, −0.028]
Indirect (Stressors → Symptoms)	0.013	0.010	0.008	1.659	0.097	[0.000, 0.029]
Total (Coping strategies → Symptoms)	−0.081	−0.055	0.028	−2.906	0.004	[−0.135, −0.028]
Total (Stressors → Symptoms)	0.754	0.600	0.042	17.831	< 0.001	[0.667, 0.836]

B = unstandardized coefficient; β = standardized coefficient; SE = standard error; CI = 95% confidence interval.

## 4 Discussion

The present study identified that 44.2% of students reported high levels of academic stress symptoms and 55.6% reported high levels of stressors. These results are consistent with the findings of 43 studies conducted during the pandemic among university students, with a prevalence trend ranging from 27 to 42%, depending on the socioeconomic conditions of each context (Peng et al., 2022). Similarly, a study conducted in the province of Madre de Dios reported a prevalence of 47% for this variable at the beginning of the pandemic (Estrada et al., 2021), in a geographic area with a high mortality rate (97 deaths per 100,000 inhabitants) due to COVID-19 (Flores et al., 2021). Regarding coping strategies, this study found that 65.5% of students reported high levels. In this regard, it is important to note that no prevalence findings expressed as percentages have been identified for this factor; rather, it has been examined in relation to the identification of problem-focused and emotion-regulation strategies as the most frequently used during the pandemic period (Cuchula, 2025).

High academic engagement was observed in 42.3% of the sample, a lower proportion than the 54.7% reported among Cuban nursing students during the pandemic (García et al., 2022). This difference may be linked to the experience of health sciences students in relation to their future professional role and the emergency health conditions, which may have stimulated their vocational orientation toward service and their interest in training. Similarly, an evaluation conducted with Mexican students from different professional fields showed that the highest percentage fell within the moderate to intermediate levels of engagement and increased progressively across different stages of the pandemic between 2020 and 2022 (Hernández and Lerma, 2025). A different finding in Peruvian students indicated that more than 50% were at the low level (Zavaleta et al., 2021). Together, these results underscore the importance of contextual differences.

Significant sex-based differences were found, with women reporting higher levels of stressors and stress symptoms than men, whereas no significant differences were observed for coping strategies. These results are similar to those reported for stress in university students from Peru and Mexico, especially in relation to stressors and symptoms (Estrada et al., 2021; Moreno-Treviño et al., 2022). Women also showed a greater tendency to seek support as a coping strategy for stressors (Scafarelli and García, 2010; Cabanach et al., 2013). In terms of academic engagement, the pattern was similar, as women showed a greater predisposition to prioritize their academic training, reporting higher dedication, vigor, and absorption than men. This is consistent with previous research in Spanish university students (Pérez et al., 2018), in contrast to a study in the Peruvian population that did not detail such differences (Arias-Chávez et al., 2020).

Findings by age showed significant differences, particularly among students aged 21–24 years, an age group considered to have lower tolerance to stressors and greater symptomatology in a study conducted with students in Huancayo, Peru (Castro and Millan, 2024). This is consistent with life-stage conditions associated with young adults aged 20 to 25 years, who face stress because of age-related responsibilities and the challenge of developing affective and economic autonomy (García-Ross et al., 2012; Marzana et al., 2010). No significant differences were found for academic engagement overall or for the vigor and absorption dimensions, which suggests relative stability in this regard, although scores were higher among students aged 25 years and older because of the responsibility and maturity associated with this stage (Córdova-Rojas et al., 2023).

Regarding difficulties accessing virtual classes, no significant differences were observed in stress-related factors or academic engagement, although students who reported access barriers tended to show higher scores for both stress and engagement than those who did not. These findings suggest that challenging and limiting conditions can generate strain while also activating adaptive responses that help individuals cope with the situation. From this perspective, engagement and persistence may be understood as resources for achieving or restoring a state of homeostasis, a view associated with resilience under adverse conditions (Mosanya, 2021).

When participants were grouped by perceived learning achievement (limited or sufficient), significant differences were observed in the variables assessed, with higher ranks in stress-related factors and lower ranks in academic engagement among students who reported limited learning achievement. These results are consistent with the direct relationship between the perception of academic failure and stress found in students in Hong Kong (Wing-Sze, 2017). They are also associated with the fact that low perceived academic achievement reduces willingness to learn and weakens students’ engagement with their academic activities (Ormrod, 2005).

Regarding the degree program, no significant differences in stress were found between psychology and engineering students, with respect to stress-related factors, which is similar to findings reported among Peruvian students in three specializations within education programs (Estrada et al., 2021), but contrasts with findings in German students, where health sciences students reported lower stress than computer science students (Olson et al., 2025). With respect to academic engagement, no specific comparative studies were identified across different specializations or degree programs.

It is also important to consider students’ occupational status, that is, whether they devote themselves exclusively to studying or also engage in paid work. In this regard, significant differences favored students who combined study with paid work, who showed greater use of coping strategies and higher academic engagement (vigor and dedication) than students who only studied. These results are supported by previous studies on stress and occupational status among Peruvian university students (Tantalean-Terrones et al., 2024). However, no prior studies were identified that compared academic engagement across different occupational statuses.

The results indicate a significant moderate positive correlation between academic engagement and its dimensions and the strategies factor, together with a weak negative correlation with the stress symptoms factor. In this regard, the study by Extremera et al. (2007) shows that perceived stress—especially the recognition of signs and symptoms of stress—correlates negatively with absorption, vigor, and dedication. In other words, the higher the perceived stress, the less favorable the conditions for sustaining academic engagement, a finding similar to that reported in German students, where a high and significant correlation was observed (Olson et al., 2025). Likewise, a positive correlation was found between coping strategies and the dimensions of academic engagement, which is theoretically consistent, although no comparison studies were identified. Regarding the stressors dimension, no correlation was found with any of the engagement dimensions. This may be because vigor, dedication, and absorption are intrinsic to the individual’s experience, whereas stressors are circumstantial and external.

The proposed model separates the factors included in the SISCO V Academic Stress Inventory (Adapted), based on the framework of Lazarus and Folkman (1984) and Folkman (2013), which assesses stressful events in relation to the use of specific coping strategies (problem-focused coping or management of emotional distress) and their associations with symptoms (the physical and mental manifestations of stress). These three elements are linked to overall academic engagement and its dimensions in a model in which stress symptoms were specified as the endogenous variable, stressors and coping strategies as exogenous variable, and academic engagement as a mediator. In this sense, the findings suggest that stressors, coping strategies, and academic engagement were jointly associated 37.5% with variance in stress symptoms, and that academic engagement was negatively associated with stress symptoms within the model.

To our knowledge, no previous studies have used this version of the instrument in an academic context adapted to the pandemic, nor have previous studies analyzed the separation of its components for the development of structural models. However, the contribution of stress-coping strategies to academic engagement is noteworthy, given its positive implications for the performance of university students in education and other disciplines (Rabal and Gonzales, 2023; Vizoso et al., 2018). Regarding the potential mediating role of academic engagement, this pattern is partially consistent with evidence from a study of U. S. university students in which this variable mediated the relationship between stress and academic performance (Tormon et al., 2023).

## 5 Limitations and future perspectives

The limitations of the study are related to the period in which the research was conducted and to the use of an instrument adapted to the context of virtual learning during the pandemic for the assessment of academic stress; this reduces the possibility of strictly replicating the results in post-pandemic scenarios, in which virtual learning plays a complementary role in higher education. In addition, the instrument has only one study in which its construct validity was not established through confirmatory factor analysis, which is why such an analysis had to be conducted in the present study to address this limitation.

It should be noted that the use of nonprobability snowball sampling may have introduced selection bias, homogenized the sample, and compromised its statistical representativeness. In this regard, the findings reflect trends observed in the sample under study, but do not constitute estimates that can be generalized to the Peruvian university population. Likewise, the structural equation model was limited to the evaluation of direct, indirect, and total relationships among the variables and does not establish conclusive causal inferences because the study used a cross-sectional design.

Limitations related to the use of self-report instruments are also acknowledged because such measures may affect the precision of responses due to social desirability and subjective perception, even though confidentiality and anonymity were guaranteed. Finally, online data collection does not guarantee the absence of bias in item comprehension because participants did not have immediate support available to resolve possible doubts while completing the scales.

The analysis of academic stress and academic engagement opens up a wide range of possibilities for the development of both cross-sectional and longitudinal research. In cross-sectional studies, this includes the incorporation of mediating variables such as emotional regulation, academic self-efficacy, perceived social support, and digital competencies, among other sociodemographic factors that may support logistic regression analyses and neural network models. These approaches would allow researchers to propose structural models that provide a broader understanding of the educational environment, from basic education to higher education. In longitudinal studies, in turn, they facilitate the identification of students’ trajectories and developmental changes, particularly in online learning contexts, where analyses may also incorporate the quality of learning platforms, student-teacher interaction, academic workload, among other factors.

## 6 Conclusion

The present study highlights the value of academic engagement as a variable that contributes to students’ personal and professional development and their preparation for professional life under conditions that affect their well-being during the educational process. It also underscores the need to expand its study in relation to stress and other psychological and sociodemographic conditions in order to develop structural models that clarify its origin and the factors that strengthen or weaken it. This knowledge is essential for designing public policies that support effective interventions to promote educational retention and academic success among students at different educational levels, with implications for individual and community social development.

## Data Availability

The original contributions presented in the study are included in the article/Supplementary material, further inquiries can be directed to the corresponding author/s.
