# Supplementary material for: Academic stress and academic engagement among Peruvian university students during their first experience with online learning
Source: Front Psychol. 2026 Jul 16;17:1859567. doi: 10.3389/fpsyg.2026.1859567 (PMC13422203; doi:10.3389/fpsyg.2026.1859567)
Supplement: Supplementary file 1 [file Table_1.DOCX]

**Supplementary Tables**

**SUPPLEMENTARY TABLE A Normality test for the variables of stress and academic engagement**

| Variable/ Dimension | *K-S* | *gl* | *Sig.* | Normality | Test |
| --- | --- | --- | --- | --- | --- |
| Stressors | ,068 | 527 | ,000 | They do not comply | Spearman's rho |
| Symptoms | ,053 | 527 | ,001 |  |  |
| Coping strategies | ,055 | 527 | ,001 |  |  |
| Academic engagement | ,054 | 527 | ,001 | They do not comply |  |
| Vigor | ,077 | 527 | ,000 |  |  |
| Dedication | ,115 | 527 | ,000 |  |  |
| Absorption | ,052 | 527 | ,002 |  |  |

Supplementary Table A presents the results of the Kolmogorov–Smirnov normality test for the study variables and dimensions. The findings showed that stressors, symptoms, coping strategies, academic engagement, vigor, dedication, and absorption did not meet the assumption of normality (p < .05 in all cases). Therefore, nonparametric analyses were performed using Spearman’s rho correlation coefficient.

**SUPPLEMENTARY TABLE B Normality test for the Variables of Stress and Academic Engagement by age**

| Variable/ Dimension | Age groups | *K-S* | *gl* | *Sig.* | Test |
| --- | --- | --- | --- | --- | --- |
| Stressors | 17 - 20 | ,098 | 99 | ,020 | Kruskal-Wallis |
|  | 21 - 24 | ,071 | 305 | ,001 |  |
|  | 25 - + | ,078 | 123 | ,061 |  |
| Symptoms | 17 - 20 | ,062 | 99 | ,200^*^ | Kruskal-Wallis |
|  | 21 - 24 | ,062 | 305 | ,006 |  |
|  | 25 - + | ,058 | 123 | ,200^*^ |  |
| Coping strategies | 17 - 20 | ,087 | 99 | ,063 | Kruskal-Wallis |
|  | 21 - 24 | ,057 | 305 | ,018 |  |
|  | 25 - + | ,060 | 123 | ,200^*^ |  |
| Academic engagement | 17 - 20 | ,082 | 99 | ,101 | Kruskal-Wallis |
|  | 21 - 24 | ,055 | 305 | ,024 |  |
|  | 25 - + | ,068 | 123 | ,200^*^ |  |
| Vigor | 17 - 20 | ,086 | 99 | ,067 | Kruskal-Wallis |
|  | 21 - 24 | ,067 | 305 | ,002 |  |
|  | 25 - + | ,086 | 123 | ,026 |  |
| Dedication | 17 - 20 | ,122 | 99 | ,001 | Kruskal-Wallis |
|  | 21 - 24 | ,106 | 305 | ,000 |  |
|  | 25 - + | ,162 | 123 | ,000 |  |
| Absorption | 17 - 20 | ,096 | 99 | ,025 | Kruskal-Wallis |
|  | 21 - 24 | ,070 | 305 | ,001 |  |
|  | 25 - + | ,078 | 123 | ,063 |  |

Supplementary Table B presents the results of the Kolmogorov–Smirnov normality test for stress and academic engagement variables according to age groups. The overall distributional pattern indicated that the assumption of normality was not consistently met across groups. Therefore, nonparametric comparisons were conducted using the Kruskal–Wallis test.

**SUPPLEMENTARY TABLE C Normality test for the Variables of Stress and Academic Engagement by age by sex**

| Variable/ Dimension | Sex | *K-S* | *gl* | *Sig.* | Test |
| --- | --- | --- | --- | --- | --- |
| Stressors | Male | ,089 | 175 | ,002 | **U de Mann-Whitney** |
|  | Female | ,073 | 352 | ,000 |  |
| Symptoms | Male | ,064 | 175 | ,077 | **U de Mann-Whitney** |
|  | Female | ,059 | 352 | ,005 |  |
| Coping strategies | Male | ,075 | 175 | ,018 | **U de Mann-Whitney** |
|  | Female | ,056 | 352 | ,009 |  |
| Academic engagement | Male | ,046 | 175 | ,200* | **U de Mann-Whitney** |
|  | Female | ,069 | 352 | ,000 |  |
| Vigor | Male | ,089 | 175 | ,002 | **U de Mann-Whitney** |
|  | Female | ,082 | 352 | ,000 |  |
| Dedication | Male | ,101 | 175 | ,000 | **U de Mann-Whitney** |
|  | Female | ,131 | 352 | ,000 |  |
| Absorption | Male | ,090 | 175 | ,002 | U de Mann-Whitney |
|  | Female | ,059 | 352 | ,005 |  |

As shown in Supplementary Table C, the Kolmogorov–Smirnov test indicated non-normal distributions for most stress and academic engagement variables across sex groups. Accordingly, the Mann–Whitney U test was used for group comparisons

**SUPPLEMENTARY TABLE D Normality test for the Variables of Stress and Academic Engagement by age by major**

| Variable/ Dimension | Major | *K-S* | *gl* | *Sig.* | Test |
| --- | --- | --- | --- | --- | --- |
| Stressors | Engineering | ,054 | 245 | ,084 | **U de Mann-Whitney** |
|  | Psychology | ,060 | 282 | ,015 |  |
| Symptoms | Engineering | ,068 | 245 | ,007 | **U de Mann-Whitney** |
|  | Psychology | ,076 | 282 | ,000 |  |
| Coping strategies | Engineering | ,064 | 245 | ,018 | **U de Mann-Whitney** |
|  | Psychology | ,049 | 282 | ,097 |  |
| Academic engagement | Engineering | ,055 | 245 | ,075 | **U de Mann-Whitney** |
|  | Psychology | ,065 | 282 | ,005 |  |
| Vigor | Engineering | ,076 | 245 | ,002 | **U de Mann-Whitney** |
|  | Psychology | ,079 | 282 | ,000 |  |
| Dedication | Engineering | ,112 | 245 | ,000 | **U de Mann-Whitney** |
|  | Psychology | ,115 | 282 | ,000 |  |
| Absorption | Engineering | ,058 | 245 | ,048 | U de Mann-Whitney |
|  | Psychology | ,056 | 282 | ,033 |  |

As shown in Supplementary Table D, the Kolmogorov–Smirnov test indicated non-normal distributions for several stress and academic engagement variables across Engineering and Psychology groups (p < .05 in multiple cases). Accordingly, the Mann–Whitney U test was used for group comparisons.

**SUPPLEMENTARY TABLE E Normality test for the Variables of Stress and Academic Engagement by age by occupation**

| Variable/ Dimension | Occupation | *K-S* | *gl* | *Sig.* | Test |
| --- | --- | --- | --- | --- | --- |
| Stressors | Study | ,076 | 335 | ,000 | **U de Mann-Whitney** |
|  | Study and work | ,119 | 192 | ,000 |  |
| Symptoms | Study | ,061 | 335 | ,005 | **U de Mann-Whitney** |
|  | Study and work | ,046 | 192 | ,200^*^ |  |
| Coping strategies | Study | ,068 | 335 | ,001 | **U de Mann-Whitney** |
|  | Study and work | ,059 | 192 | ,096 |  |
| Academic engagement | Study | ,060 | 335 | ,006 | **U de Mann-Whitney** |
|  | Study and work | ,051 | 192 | ,200^*^ |  |
| Vigor | Study | ,075 | 335 | ,000 | **U de Mann-Whitney** |
|  | Study and work | ,077 | 192 | ,007 |  |
| Dedication | Study | ,116 | 335 | ,000 | **U de Mann-Whitney** |
|  | Study and work | ,129 | 192 | ,000 |  |
| Absorption | Study | ,066 | 335 | ,001 | U de Mann-Whitney |
|  | Study and work | ,080 | 192 | ,004 |  |

As shown in Supplementary Table E, the Kolmogorov–Smirnov test indicated non-normal distributions for several variables across occupational groups (study only vs. study and work). Accordingly, the Mann–Whitney U test was used for group comparisons.

**SUPPLEMENTARY TABLE F Normality test for the Variables of Stress and Academic Engagement by age by difficulty accessing online classes**

| Variable/ Dimension | Access | *K-S* | *gl* | *Sig.* | Test |
| --- | --- | --- | --- | --- | --- |
| Stressors | No difficulty | ,092 | 116 | ,018 | **U de Mann-Whitney** |
|  | Difficulty | ,063 | 411 | ,001 |  |
| Symptoms | No difficulty | ,098 | 116 | ,008 | **U de Mann-Whitney** |
|  | Difficulty | ,047 | 411 | ,030 |  |
| Coping strategies | No difficulty | ,075 | 116 | ,136 | **U de Mann-Whitney** |
|  | Difficulty | ,059 | 411 | ,001 |  |
| Academic engagement | No difficulty | ,076 | 116 | ,106 | **U de Mann-Whitney** |
|  | Difficulty | ,053 | 411 | ,007 |  |
| Vigor | No difficulty | ,058 | 116 | ,200^*^ | **U de Mann-Whitney** |
|  | Difficulty | ,092 | 411 | ,000 |  |
| Dedication | No difficulty | ,145 | 116 | ,000 | **U de Mann-Whitney** |
|  | Difficulty | ,122 | 411 | ,000 |  |
| Absorption | No difficulty | ,062 | 116 | ,200^*^ | U de Mann-Whitney |
|  | Difficulty | ,055 | 411 | ,005 |  |

As shown in Supplementary Table F, the Kolmogorov–Smirnov test indicated non-normal distributions for several stress and academic engagement variables across levels of difficulty accessing online classes. Accordingly, the Mann–Whitney U test was used for group comparisons.

**SUPPLEMENTARY TABLE G Normality test for the Variables of Stress and Academic Engagement by age by learning outcomes**

| Variable/ Dimension | Learning | *K-S* | *gl* | *Sig.* | Test |
| --- | --- | --- | --- | --- | --- |
| Stressors | No achievements | ,069 | 199 | ,023 | **U de Mann-Whitney** |
|  | With achievements | ,085 | 328 | ,000 |  |
| Symptoms | No achievements | ,068 | 199 | ,026 | **U de Mann-Whitney** |
|  | With achievements | ,062 | 328 | ,004 |  |
| Coping strategies | No achievements | ,053 | 199 | ,200^*^ | **U de Mann-Whitney** |
|  | With achievements | ,064 | 328 | ,003 |  |
| Academic engagement | No achievements | ,049 | 199 | ,200^*^ | **U de Mann-Whitney** |
|  | With achievements | ,064 | 328 | ,002 |  |
| Vigor | No achievements | ,086 | 199 | ,001 | **U de Mann-Whitney** |
|  | With achievements | ,085 | 328 | ,000 |  |
| Dedication | No achievements | ,083 | 199 | ,002 | **U de Mann-Whitney** |
|  | With achievements | ,128 | 328 | ,000 |  |
| Absorption | No achievements | ,076 | 199 | ,007 | U de Mann-Whitney |
|  | With achievements | ,054 | 328 | ,023 |  |

As shown in Supplementary Table G, the Kolmogorov–Smirnov test indicated non-normal distributions for several stress and academic engagement variables across learning outcome groups. Accordingly, the Mann–Whitney U test was used for group comparisons.

**SUPPLEMENTARY TABLE H Fit indices for academic stress and engagement scales**

| Scale | *CMIN* | *df* | *CMIN/df* | *PClose* | *CFI* | *TLI* | *RMSEA​* | *SRMR* |
| --- | --- | --- | --- | --- | --- | --- | --- | --- |
| SISCO 47 (Adapted) | 4343.799 | 1028 | 4.225 | .000 | 0.960 | 0.958 | 0.078 | 0.070 |
| UWES-17 | 1007.360 | 116 | 8.684 | .000 | 0.924 | 0.911 | 0.121 | 0.059 |

As shown in Supplementary Table H, the SISCO 47 (adapted) scale showed an acceptable model fit (CFI = 0.960; TLI = 0.958; RMSEA = 0.078), whereas the UWES-17 showed a weaker fit (CFI = 0.924; TLI = 0.911; RMSEA = 0.121), despite an acceptable SRMR (0.059). Overall, the SISCO 47 (adapted) exhibited better fit indices than the UWES-17.

**SUPPLEMENTARY TABLE I Convergent and discriminant validity of the academic stress and engagement scales**

| Variables | Dimension | *AVE* | Heterotrait-monotrait ratio | | | |
| --- | --- | --- | --- | --- | --- | --- |
|  |  |  | Factor 1 | | Factor 2 | Factor 3 |
| SISCO 47 (Adapted) | Symptoms | 0.421 | 1.000 | |  |  |
|  | Stressors | 0.525 | 0.664 | | 1.000 |  |
|  | Coping strategies | 0.309 | 0.141 | | 0.147 | 1.000 |
| UWES-17 | Vigor | 0.487 | | 1.000 |  |  |
|  | Dedication | 0.736 | | 0.823 | 1.000 |  |
|  | Absorption | 0.512 | | 1.012 | 0.874 | 1.000 |

As shown in Supplementary Table I, convergent validity (AVE) was adequate for some dimensions (e.g., stressors and dedication), while others fell below the recommended threshold (e.g., coping strategies and symptoms). Discriminant validity (HTMT) was generally acceptable, although some values among UWES-17 dimensions suggested partial overlap. Overall, the scales showed mixed but acceptable validity evidence.

**SUPPLEMENTARY TABLE J Reliability of the scales measuring stress and academic engagement**

| Academic stress | ω | α | Academic engagement | ω | α |
| --- | --- | --- | --- | --- | --- |
| Symptoms | 0.830 | 0.826 | Vigor | 0.943 | 0.895 |
| Stressors | 0.917 | 0.913 | Dedication | 0.954 | 0.928 |
| Coping strategies | 0.838 | 0.839 | Absorption | 0.815 | 0.810 |
| Total | 0.942 | 0.939 | Total | 0.919 | 0.900 |

As shown in Supplementary Table J, both the academic stress and academic engagement scales demonstrated good to excellent reliability, with omega and alpha coefficients above 0.80 across all dimensions and high overall reliability for both scales.
